# Organogel-nanoemulsion containing nisin and D-limonene and its antimicrobial activity

**DOI:** 10.3389/fmicb.2015.01010

**Published:** 2015-09-22

**Authors:** Weiya Bei, Yan Zhou, Xuya Xing, Mohamed Reda Zahi, Yuan Li, Qipeng Yuan, Hao Liang

**Affiliations:** State Key Laboratory of Chemical Resource Engineering, Beijing University of Chemical TechnologyBeijing, China

**Keywords:** D-limonene, nisin, organogel-nanoemulsion, antimicrobial activity, food preservation

## Abstract

The aim of this study was to investigate a novel delivery system containing D-limonene and nisin by food organogel-nanoemulsion and study its effect on the antimicrobial activity. Organogel-nanoemulsion containing with D-limonene and nisin or without nisin was prepared by a homogenization method. Factors that may affect the droplet size and stability of organogel-nanoemulsion such as pressure and surfactant to oil ratio (SOR) were studied. The average droplet size decreased with pressure, and the organogel-nanoemulsion could achieve good stability at low SOR. Positive effects and outstanding antimicrobial activities of organogel-nanoemulsion containing with D-limonene and nisin were confirmed by minimal inhibitory concentrations comparison, growth curves of bacteria, scanning electron microscopy and determination of cell constituents’ release. Furthermore, the organogel-nanoemulsion applied as food preservative in milk also shown excellent antimicrobial performance. Overall, the research described in the current article show that organogel-nanoemulsion containing with D-limonene and nisin may be an effective antimicrobial system for the production and preservation of food.

## Introduction

It is well known that microbial contamination has got wide attention, due to it may bring potential adverse effects to consumers (Oliveira et al., 2011). Under the impacts of globalization and quick distribution systems, these can have good impacts with profound and even fatal result (Ellis et al., 2012). Some natural antimicrobial substances were used jointly with new technologies to resolve the issues of pathogenic bacteria, which could improve food safety and product quality (Gálvez et al., 2010). Antimicrobial delivery systems were proposed as potential solutions to improve effectiveness of antimicrobials in food matrices by safeguarding antimicrobials from contacting food matrix components and releasing them incessantly (Xiao et al., 2011). D-limonene (4-isopropenyl-1-methylcyclohexene) is an ingredient of many citrus fruits and it is widely used in many fields, including cosmetics, foods, and other products. A number of researchers have proved that D-limonene had bactericide, antioxidant, chemo-preventative, and therapeutic activities (Gálvez et al., 2010). Its outstanding antimicrobial activities have already been proven against different species of food-related microorganisms, such as *Staphylococcus aureus*, *Listeria monocytogenes*, *Salmonella enterica*, *Saccharomyces bayanus* (Abi-Ayada et al., 2011).

However, due to high hydrophobic and easy to oxidative degradation natures of D-limonene, it is difficult in achieving an even dispersion in water and directly results in its loss of activity. It will require more D-limonene to get identical antimicrobial efficiencies in foods, especially in the areas such as the interface of two different phases (Zhang et al., 2014). Therefore, it is very important and necessary protect D-limonene from chemical degradation and improve its water-solubility.

A number of approaches have been explored to improve these shortcomings and limitations of hydrophobic, oxidation-prone biologically active compounds. Many research groups have focused on combinations of essential oils (EOs) with synergistic activity (Gutierrez et al., 2009), and the combinations of EOs with other natural antimicrobial(e.g., Nisin) could get effective antibacterial activity with a small amount, then the effect of negative sensory on foods can be mitigated significantly (Govaris et al., 2010). Nisin is a peptide, the product of some Lactococcus lactis subsp. It was introduced for the first time in the UK as a food preservative. It has been widely acknowledged and applied to daily use in many countries (Delves-Broughton et al., 1996). In addition, other research groups found that emulsification was also a good way to improve their solubilities and stabilities (Benjamin et al., 2012). In our previous work, it was found that D-limonene nanoemulsion with nisin prepared by catastrophic phase inversion (CPI) method have shown good stability and outstanding antimicrobial activity (Zhang et al., 2014). So on this occasion, we studied organogel-nanoemulsion containing with D-limonene and nisin on the basis of early research.

Organogels are semi-solid systems, including liquid oil trapped within a three-dimensional networked structure which is formed by the self-assembly of a low concentration of organogelator molecules in a variety of organic liquids (Hughes et al., 2009). Organogel technology has been applied in many industries, and its potentials continue to be developed. Hence, we know that organogels have bright future in the industrial applications, especially in food industry, for its ability to structure edible oils. To our best knowledge, there are no reports about the exploration of the impacts of nisin on the antibiosis effect of D-limonene organogel-nanoemulsion for achieving a better preservative effect, which is the purpose of this research. In addition, we also focus on developing a novel antimicrobial delivery system combining positive effect of these two antibacterial agents at the same time.

## Materials and Methods

### Materials

D-limonene was obtained from Florida Worldwide Citrus Products Group Inc. (Bradenton, FL, USA). Nisin (10^4^ IU/mL) was purchased from Lanzhou Weiri Bio-Engineering Co., Ltd., (Lanzhou, China). Sucrose stearate; sorbitan monooleate (Tween80), glutaraldehyde, sodium chloride (purity > 99.5%), kanamycin sulfate was supplied by the Sinopharm Chemical Reagent Co., Ltd. (Beijing, China). Deionized water was filtered prior to use. Peanut oil and fresh 2% reduced fat milk were purchased from supermarket.

### Preparation of Organogel-Nanoemulsions Containing D-limonene and Nisin

Nisin (180 mg) was dissolved in 3 mL deionized water as the aqueous phase. Oil phase (10 g) containing stearic acid (7 g), sucrose stearate 170 (S170; 5% w/w), peanut oil (88 or 68% w/w) and D-limonene (0 or 20% w/w) were heated and stirred (80°C), until the solution was clear. Then the two phases were homogenized (HENC homogenizer, Shanghai, China), and then frozen and lyophilized (FD-1C-50, Beijing, China), to produce an organogel phase. Next, the prepared organogel phase (10 g) was placed in an 80°C water bath again to form a transparent oil phase, and dispersed into another water phase (50 mL) containing Tween 80 with stirring, followed by high pressure homogenization to form nanoemulsion.

### Measurement of Droplet Size Diameters

Dynamic light scattering was used to measure the mean particle diameters of samples at 25°C (Zetasizer Nano-ZS90, Malvern Instruments, Malvern, UK).

### Antimicrobial Activity

#### Microbial Strains and Growth Conditions

Three food-related microorganisms included the Gram-positive bacteria *S. aureus* ATCC6538, and *Bacillus subtilis* ATCC6633, the Gram-negative bacteria *Escherichia coli* ATCC8739. All strains were supplied by China General Microbiological Culture Collection Center (Beijing, China), maintained at 4°C and incubated at 37°C (Zhang et al., 2014).

#### Synergism Testing: Checkerboard Method

The interactive inhibition of antimicrobial compounds *in vitro* was evaluated by broth dilution checkerboard method (Hemaiswarya et al., 2008; Zhang et al., 2014). D-limonene was diluted twofold in vertical orientation, while nisin was diluted twofold in horizontal orientation. Their respective concentrations were prepared corresponding to ½, ¼, and ⅛ of the minimal inhibitory concentration (MIC) values, respectively. Next, 400 μL suspension containing (1 × 10^8^ CFU/mL) of the indicator microorganism was added to each tube and incubated overnight at 37°C.

We calculated the fractional inhibitory concentration indices (FICI) as follow: FICI = FICA + FICB, where FICA = (MICA of the combination/MICA alone) and FICB = (MICB of the combination/MICB alone). The results were divided into the following categories: synergy (FICI ≤ 0.5), addition (0.5 ≤ FICI ≤ 1), indifference (1 ≤ FICI ≤ 4) or antagonism (FICI > 4) (Zhang et al., 2014).

#### Determination of Minimal Inhibitory Concentration

The double broth dilution method was adopted to confirm the MIC values with some modifications as described by previous researchers (Weerakkody et al., 2010). After adding appropriate antimicrobial agents to the first tube containing 4 mL of broth, serial twofold dilutions were made. A 400 μL suspension (1 × 10^8^ CFU/mL) of designated microorganism was added to each tube.

The tube contained only broth and microorganism as a negative control. Meanwhile, the tube contained broth, 50 μg/mL of kanamycin sulfate and microorganism as a positive control (Al-Reza et al., 2009; Lv et al., 2011).

#### Growth Curves of Bacterium Treated with Organogel-Nanoemlusion

Logarithmic phase cells were utilized in the tests, following by a dilution to prepare bacterium suspensions of 10^5^–10^6^ CFU/mL. The suspensions were dealt with different concentrations of organogel-nanoemulsions of D-limonene and nisin (control, MIC, 2 × MIC and 4 × MIC), then incubated in same suitable conditions. Cells were harvested by centrifugation (5000 rpm for 10 min) every 2 h. After three times of washing and collection, cells were re-suspended in phosphate buffer solution (PBS, 0.1 M, pH 7.0). The quantity of cells was measured by UV-Vis (SHIMADZU UV2450) (Govaris et al., 2010; Zhang et al., 2014).

### Scanning Electron Microscopy (SEM) Analysis

Scanning electron microscopy (SEM) studies were adopted as reported by Moosavy et al. (2008) and Zhang et al. (2014) with some modifications. Three tested microorganisms (about 1 × 10^8^ CFU/mL), which in logarithmic growth phase, were treated with each MIC of organogel-nanoemlusion containing D-limonene and nisin. Then samples were incubated at room temperature for 3 h, and harvested by centrifugation and washed twice with PBS (0.1 M, pH 7.0). And then resuspended in PBS including 2.5% glutaraldehyde and kept for 2h at -4°C. Followed by further dehydrated at different ethanol concentrations. At last, the thalli were fixed and sputter-coated with gold, then observed by SEM (Zeiss Supra^7M^ 55, Stuttgart, Germany) (Lv et al., 2011; Zhang et al., 2014).

### Determination of the Release of Cell Constituents

The release of cell constituents was inspected through the method previously described (Rhayour et al., 2003; Lv et al., 2011; Zhang et al., 2014). Individual cultures were grown for 24 h in their respective medium, after which 100 mL was collected and centrifuged, washed three times with PBS, and finally resuspended in PBS (0.1 M, pH 7.0). The washed cell suspensions were incubated at 37°C in an environmental incubator shaker for 1 h after treated by different concentration of organogel-nanoemulsion (control, MIC and 2 × MIC). Then, 2 mL of samples were collected and centrifuged, and the centrifuged cell supernatant was used to measure UV absorption at 260 nm.

### Determination of Antimicrobial Activity of Oraganogel-Nanoemulsions with Nisin and D-limonene in Milk

In the test, fresh 2% reduced fat milk was chosen as the target food to assess antimicrobial performance of organogel-nanoemusions with D-limonene and nisin. The milk samples were incubated at room temperature for 5 days in the presence of different conditions of organogel-nanoemulsions (control and 2 × MIC). After incubating the mixtures at room temperature for 120 h, the pour plate method was used to enumerate the viable bacteria (Ma et al., 2013).

### Statistical Analysis

All experiments were performed in triplicate. The data were recorded as mean ± standard deviation for the measurements. Differences between means were considered significant at *p* < 0.05. The SPSS programmer (SPSS, version 12.0 for Windows, SPSS Inc.) was used to analyze the results.

## Results

### Preparation and Characterizations of Organogel-Nanoemulsions with D-limonene and Nisin

**FIGURE 1:**
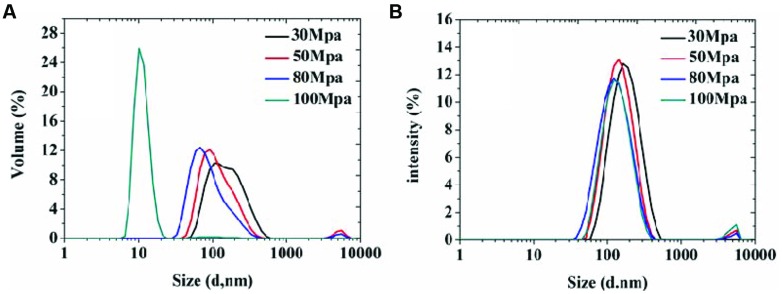
**The particle size distributions of organogel-nanoemulsion prepared under different homogenizing pressures were analyzed by DLS [size distributions by volume **(A)** and intensity **(B)**]**.

In order to achieve homogeneous and stable organogel-nanoemulsions, and establish the most appropriate surfactant to oil ratio (SOR) and pressure, which contribute to the formulation, size distribution and, more importantly, stability of organogel-nanoemulsions, the preparation process was optimized at first, a series of emulsion processes were prepared under different pressures and SOR values. All the nanoemulsion samples contained D-limonene and nisin of 6%, 15% (w/w). For pressure optimization, a wide range of preparation pressures (30, 50, 80, and 100 MPa) from high pressure homogenizer were utilized to explore and compare the particle size distributions. As the results shown in **Figure 1**, both Volume (%) value (**Figure 1A**) and Intensity (%) value (**Figure 1B**) of particle size distributions of all samples displayed bimodal results, in which the vast majority of the droplet population had a droplet-size distribution around 100 or 10 nm, only a small population around 10 μm. The highest homogenizing pressure of 100 MPa prepared the minimum droplet size of 10 nm by Volume (%) value (**Figure 1A**), which could provide large specific surface area and stable capacity for the following antimicrobial experiments of organogel-nanoemulsions.

**FIGURE 2:**
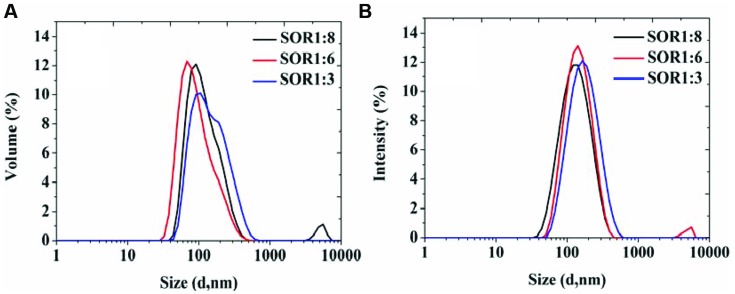
**The particle size distributions of organogel-nanoemulsion prepared under different SORs were analyzed by DLS [size distributions by volume **(A)** and intensity **(B)**]**.

During the SOR of optimization process, we chose food-grade Tween 80 as the surfactant, prepared a series of nanoemulsions with different SORs (from 1:8 to 1:3) and same homogenizing pressure of 100 MPa. The results in **Figure 2** show that all the samples exhibit similar drop size distributions with a main population around in 100 nm and a mall population in 10 nm. The results indicate that organogel-nanoemulsions prepared with very low concentration of surfactant (SOR 1:8) could achieve narrow and uniform particle size distribution, contributing to a low surfactant additions in food.

**FIGURE 3:**
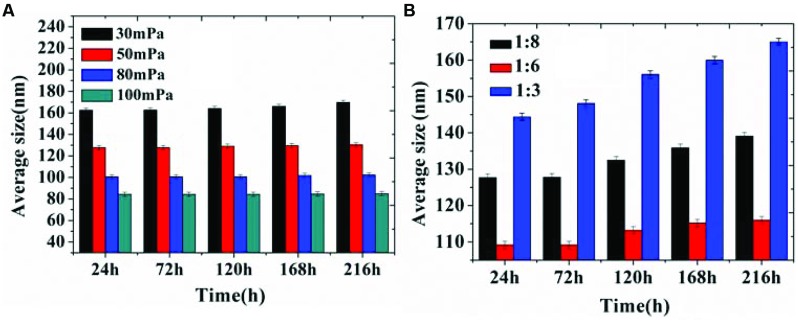
**Storage stability of organogel-nanoemulsion prepared under different pressures **(A)** and SORs **(B)** at 28°C**.

In our previous study, D-limonene organogel-based nanoemulsion exhibited a good stability: the particle size increased 10 nm at 28°C and 6 nm at 4°C during a storage period of 2 weeks (Zahi et al., 2014). In this study, we got similar and even better results. The results in **Figure 3** show that the storage stability of organogel-nanoemulsions prepared with different pressures (**Figure 3A**) and SORs (**Figure 3B**). There was only a very slight increase in the average size for all samples stored at 28°C, which confirmed that organogel-nanoemulsion containing D-limonene and nisin has an excellent stability once more. All the samples were stored at 28°C for no fewer than 3 months showed no stratification or phase separation.

### *In Vitro* Synergy between Nisin and D-limonene

To enhance the antagonistic activity and as such reduce the quantity of preservatives added to food, nisin has been found to act in synergy with various antimicrobial agents including cheaters, oregano EO, reuterin, and phage endolysin (Govaris et al., 2010). In this work, three food-related microorganisms (*S. aureus*; *B. subtilis*, and *E. coli*) were chosen as the target bacterium. When nisin and D-limonene were combined by the checkerboard method, synergistic and additive effects were observed (**Table 1**). With reference to the FICI scale, nisin and D-limonene combination displayed a synergism with regards to *S. aureus* and *B. subtilis* (FICI = 0.375), and exhibited a useful additive effect with the FICI of 1.0 for the *E. coli*. No indifference and antagonistic effects were observed (Zhang et al., 2014). The synergistic and additive effects between D-limonene and nisin could provide a strong support for the next tests of antibacterial of organogel-nanoemulsion.

**Table 1:** Fractional inhibitory concentration indices (FICI) of combinations of D-limonene and nisin against the tested microorganisms^a,b^.

Combination	*Staphylococcus aureus*		*Bacillus subtilis*		*Escherichia coli*	
	FIC	FICI	FIC	FICI	FIC	FICI
D-limonene-nisin	0.25	0.37 (S)	0.25	0.37 (S)	1.0	1.0 (A)
	0.125		0.125		0	
	5		5			

*^a^Results are means of three different experiments. ^b^The results were interpreted as synergy (S, FICI < 0.5), addition (A, 0.5 ≤ FICI ≤ 1), indifference (I, 1 < FICI ≤ 4) or antagonism (AN, FICI ≥ 4)*.

### Antimicrobial Activity of Organogel-Nanoemulsions

As the results of MIC values shown in the **Table 2**, the MICs of organogel-nanoemulsions containing both D-limonene and nisin (D-limonene 15% w/w and nisin 6% w/w) decreased obviously comparing to other two kinds of organogel-nanoemulsions containing only D-limonene (15% w/w) or only nisin (6% w/w) against all the three target microorganisms. More importantly to the Gram-negative *E. coli*, organogel-nanoemulsions with D-limonene and nisin displayed a strong enhancement (MIC 42.15 μg/mL) than the organogel-nanoemulsions with only nisin (MIC > 5000 μg/mL). As we know nisin had no antimicrobial effect on any of Gram-negative bacteria such as *E.coli*. The result maybe directly attributed to the additive effect of D-limonene with nisin in the system of organogel-nanoemulsions. Furthermore, the addition of two antibacterial agents also decreased significantly according to the MICs of D-limonene and nisin, respectively, shown in the **Table 2**, especially for the *S. aureus* (MICs of D-limonene and nisin decreased from 4.75 to 1.1 μg/mL, 0.45 to 0.33 μg/mL) and *B. subtilis* (MICs of D-limonene and nisin decreased from 4.38 to 2.2 μg/mL, 0.9 to 0.66 μg/mL).

**Table 2:** Minimal inhibitory concentrations (MIC) values of three kinds of organogel-nanoemulsions against three target microorganisms.

Microorganisms	MICs(μg/mL)		
	Organogel-nanoemulsion (D-limonene 15%)	Organogel-nanoemulsion (Nisin 6%)	Organogel-nanoemulsion (D-limonene 15% and Nisin 6%)
*S. aureus*	23.75^a^ (4.75)^b^	7.5^a^ (0.45)^b^	5.47^a^ (1.1, 0.33)^b^
*B. subtilis*	21.88 (4.38)	15 (0.9)	10.94 (2.2, 0.66)
*E. coli*	43.75 (8.75)	>5000	42.15 (8.75, 2.65)

*^a^The MICs of organogel-nanoemulsions against three target microorganisms*.

*^b^The MICs of D-limonene or/and nisin in organogel-nanoemulsions against three target microorganisms*.

### Growth Curves of Bacteria and Antimicrobial Activity in Milk

**FIGURE 4:**
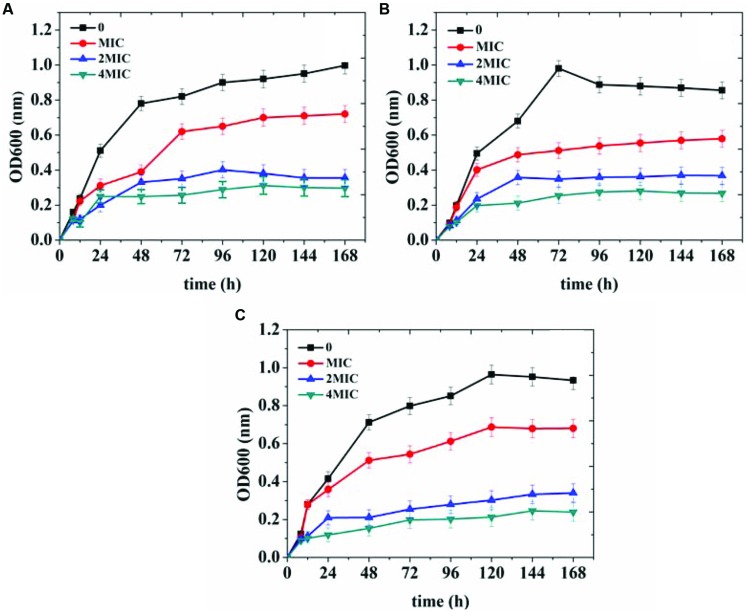
**Growth curves of *Escherichia coli***(A)**, *Staphylococcus aureus***(B),** and *Bacillus subtilis***(C)** treated with different concentrations [control, minimal inhibitory concentrations (MIC), 2 × MIC, 4 × MIC] of organogel-nanoemulsion containing D-limonene and nisin**.

In order to further verify the antibacterial effects of organogel-nanoemulsions growth curve of three target microorganisms were detected under different conditions in this study. The strains were treated with 0, MIC, 2 × MIC and 4 × MIC of organogel-nanoemulsions containing D-limonene (15% w/w) and nisin (6% w/w). After incubation for 168 h, the growth cures were summarized in **Figure 4**. As shown in **Figure 4**, organogel-nanoemulsions containing D-limonene and nisin displayed significant inhibitory effects against all the target microorganisms, especially for *E.coli* which shown the best results (**Figure 4A**). Comparing with blank samples of uncontrolled growth, organogel-nanoemulsions containing D-limonene and nisin effectively inhibited the division and reproduction of logarithmic phase of bacterium. The number of living bacterium decreased obviously under the treatments of organogel-nanoemulsions. Furthermore, the growth corves under treatment couldn’t reach the normal growth peak any more but quickly enter into decline phase, especially for the 4 × MIC which shown basically no obvious growth in number of bacterium (**Figure 4**).

**FIGURE 5:**
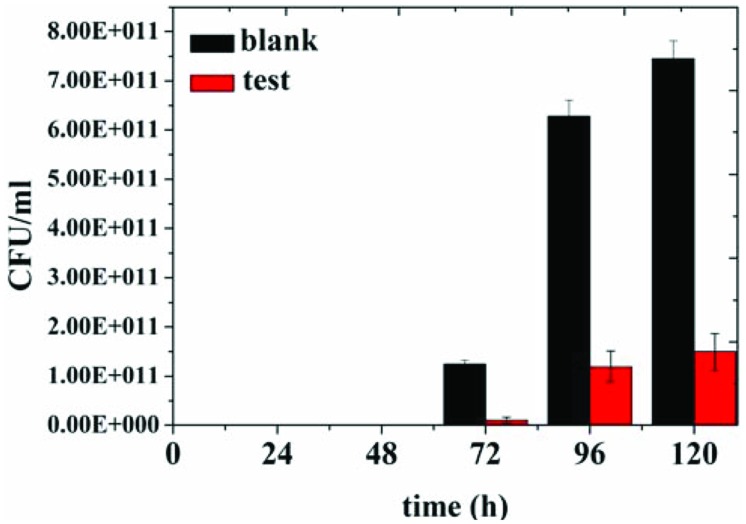
**Colony-forming units of bacteria in 2% reduced fat milk by different concentration of D-limonene organogel-nanoemulsion with nisin (control, 2 × MIC)**.

In order to assess preservative effect of the organogel-nanoemulsions with D-limonene and nisin in food, determinations of colony-forming units of bacterium in 2% reduced fat milk have been conducted under different concentrations of organogel-nanoemulsions with D-limonene and nisin (control and 2 × MIC). After incubating for 120 h at room temperature for 48 h, colony-forming units of blank and 2 × MIC treated milk samples have been calculated and presented in **Figure 5**. As shown in the **Figure 5**, blank milk sample displayed a rapid growth from 72 h and finally reached colony-forming units as many as 7.50 E + 011 CFU/mL at 120 h. However, due to the inhibition influence of 2 × MIC organogel-nanoemulsions, test milk sample shown not only slower growth rate but also limited increasing amount of units (with a final colony-forming units at 1.50 E + 011 CFU/mL around).

### Mechanisms of Action of Organogel-Nanoemulsion with Nisin and D-limonene Against Cell Membranes

**FIGURE 6:**
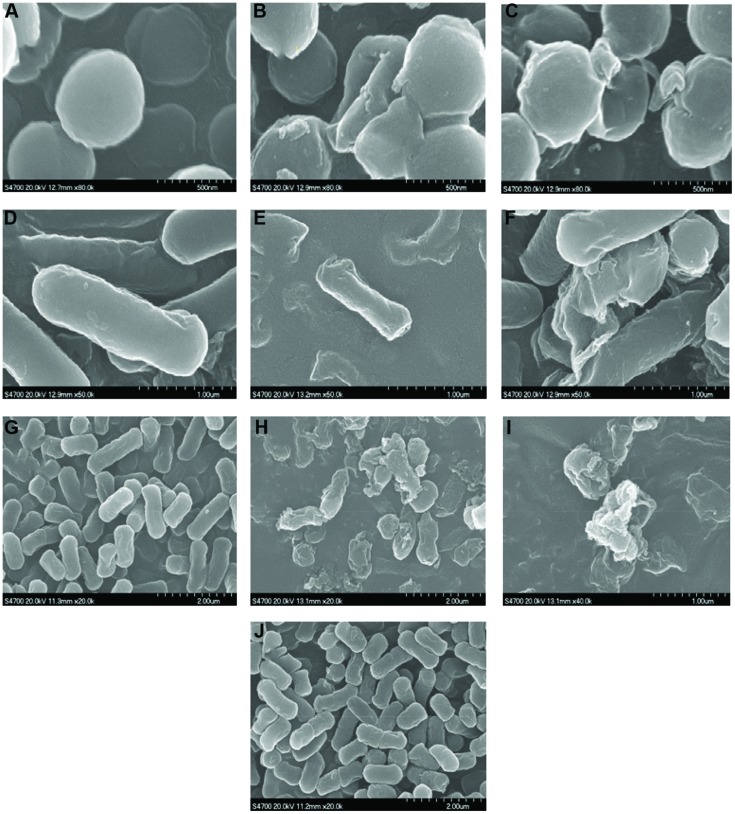
**Scanning electron micrographs of *S. aureus***(A–C)**, *B. subtilis***(D–F)**, and **(G–J)** cells: control **(A,D,G)**, treated with D-limonene organogel-nanoemulsion **(B,E,H)**, treated with D-limonene organogel-nanoemulsion with nisin **(C,F,I)**, and *E.coli* treated with organogel-nanoemulsion with nisin **(J)**, at MIC value for 3 h (magnification × 30,000 or 50,000)**.

In order to visualize the effects of D-limonene organogel-nanoemulsion with and without nisin against the cell membranes of the microbial cultures, we employed SEM of cells treated at the previously established MIC values, of D-limonene organogel-nanoemulsion (D-limonene 20% w/w), and D-limonene organogel-nanoemulsion with nisin (D-limonene 20%, nisin 6% w/w). As shown in **Figure 6**, a different degree of deformation and distortion was observed following the addition of D-limonene organogel- nanoemulsion in to the three microorganisms (**Figures 6B,E,H**). However, all microorganisms exposed to the combined treatment of D-limonene organogel-nanoemulsion with nisin suffered an almost serious collapse of the cell structure together with cell lysis (**Figures 6C,F,I**), which should be attributed to their good synergism against this Gram-negative bacteria, This can demonstrate its outstanding detrimental antimicrobial activity on the cellular integrity of all microorganisms tested. It is also noteworthy that the organogel-nanoemulsion with the inclusion of nisin had no antimicrobial effect on *E.coli* (**Figure 6J**), which is in accordance with the MIC results.

Furthermore, we measured the release of cellular content from the damaged cells as evidence that the cell membranes were damaged, and the cell constituents are released. The target microorganisms were treated with 0, MIC and 2 × MIC of D-limonene organogel-nanoemulsion with nisin and incubated for 1 h. Our results showed that the exposure to D-limonene organogel-nanoemulsion with nisin caused the release of cell constituents, which increased with the increasing concentration of D-limonene organogel-nanoemulsion with nisin as compared to the control group (**Table 3**). The most effective release of cellular constituents was observed following the 2 × MIC treatment of *B. subtilis*. The magnitude of the release of cellular constituents (**Table 3**) appears to correspond well to the observed damaged by SEM (**Figure 6**).

**Table 3:** The effect of organogel-emulsification on cell constituents′ release of the tested microorganisms.

Microorganism	Nanoemulsions concentration	Cell constituents′ release (OD_260nm_)^a^
*B. subtilis*	0	0.033 ± 0.004^b^
	MIC	0.179 ± 0.011
	MIC × 2	0.451 ± 0.008
*E. coli*	0	0.013 ± 0.003
	MIC	0.198 ± 0.012
	MIC × 2	0.314 ± 0.022
*S. aureus*	0	0.021 ± 0.004
	MIC	0.129 ± 0.009
	MIC × 2	0.337 ± 0.018

*^a^Results are presented as mean ± standard deviation from the experiments in triplicate. OD_*260nm*_, optical density at 260 nm. ^b^Means within a same strain, which are not followed by a common letter, are significantly different (*P* < 0.05)*.

## Discussion

The use of D-limonene and nisin, as antimicrobials is appealing because these compounds are a “natural” alternative to traditional treatment methods. The effectiveness of D-limonene against various foodborne pathogens has been reported in numerous studies (Gálvez et al., 2010; Abi-Ayada et al., 2011). However, D-limonene is hydrophobic and therefore has low water-solubility. The organogel-nanoemulsion process utilized in this, allowed for the dispersion of D-limonene into aqueous phases in the form of small droplets. In a water-dispersible form, D-limonene was able to act on any pathogens also present in the surrounding aqueous phase or at surfaces. And furthermore, D-limonene used in conjunction with nisin in this study.

We adopted high-pressure homogenization method to prepare organogel-nanoemulsion in this study, which is differ from CPI method that we have employed (Zhang et al., 2014). Furthermore, we incorporated D-limonene into the organogel prior to homogenization, which could immobilize the oil and improve the dispersibility of D-limonene in an easy and rapid way. Organogel-nanoemulsion was considered to combine the advantages of organogel and nanoemulsion, not only improved the solubility, but also enhanced the stability of hydrophobic compounds. Organogel-nanoemulsion can be used as a kind of excellent delivery system, especially in water-rich systems.

Droplet size is one of the most important indicators, in the assessment of emulsion performance, including the particle size and size distribution, which directly influence the stability of the emulsion and the sensory properties, function characteristics, shelf life of product (Floury et al., 2000; Benjamin et al., 2012). In this study, pressure and SOR are the main factors influencing the emulsion particle size and size distribution in the process of high pressure homogenizing. Our results demonstrated that pressure was higher, the smaller the particle size, the higher the degree of droplet refinement and distribution more uniform (**Figure 1**). Due to vast addition of surfactants in emulsion could significantly influence the quality of appearance and taste of food. So we have taken this constraint into consideration and made sure that in addition to gaining stable nanoemulsion, the SOR could be minimum at the same time. The results indicate that organogel-nanoemulsions prepared with very low concentration of surfactant (SOR 1:8) could achieve narrow and uniform particle size distribution (**Figure 2**). In addition, the prepared organogel-nanoemulsions showed outstanding storage stability (**Figure 3**). The small increase in the average size for all samples stored at 28°C is maybe due to the movement of the dispersed droplet pervaded the dispersing phase, then the chances of droplet collisions increased (Zahi et al., 2014).

Due to the results of synergistic or additive effect between D-limonene and nisin above, we prepared organogel-nanoemulsions containing these two synergistic antibacterial agents to determinate if there are any improvements of antimicrobial performance. At the same time organogel-nanoemulsions containing only D-limonene and only nisin were also prepared with same content and conditions in order to easily compare the antibacterial activity. To begin with, organogel-nanoemulsions control sample (without nisin or D-limonene) had no antimicrobial effect on any of the microorganisms tested here. The results (**Table 2**) have proved that the combination of D-limonene and nisin has improved the antimicrobial proprieties significantly. Lower MICs of antimicrobial agents not only contributes to stronger antibacterial performance of the organogel-nanoemulsions, but also result in a smaller addition to the food products when applying as preservatives.

According to the aim of this study, we also pay much attention to safety problem in food. There is a need to survey the food safety performance in the agri-food chain without performing actual microbiological analysis. European food businesses have developed and validated seven indicators and corresponding assessment grids according to an extensive microbiological assessment scheme (MAS), which provided a foundation for the food safety performance diagnosis (Tajkarimi et al., 2010). The result provides positive evidence in favors of the argument that the combined application of D-limonene organogel-nanoemulsion with the inclusion of nisin imparts an excellent antimicrobial activity when applying in food.

Although the antimicrobial effects of different EOs have been shown by various microorganisms (Al-Reza et al., 2009; Govaris et al., 2010; Tajkarimi et al., 2010; Chikhoune et al., 2013), the mechanism has not been researched at length. Chemical analysis of series of EOs showed that the primary antimicrobial active components were phenols, terpenes, aldehydes, and ketones. There is a popular belief that EOs mainly acts against the cell cytoplasmic membrane. The principal mechanisms of action of D-limonene and nisin are against the cytoplasmic membranes of microorganisms, resulting in the loss of membrane integrity; the inhibition of respiratory enzymes; and dissipation of the proton-motive force (Sikkema et al., 1995; Rhayour et al., 2003; Sun, 2007). We extended our investigation to further uncover the mechanisms that underpin the action of D-limonene based organogel-nanoemlusion with nisin. The result provides positive evidence in favor of the argument that the combined application of D-limonene organogel-nanoemulsion with the inclusion of nisin imparts an excellent antimicrobial activity.

The utilization of D-limonene organogel-nanoemulsion containing nisin prepared by high-pressure homogenization method displayed good physical stability and antimicrobial activity. In addition, growth curves of bacteria have shown that D-limonene organogel-nanoemulsion with nisin inhibits the reproduction of bacteria. SEM and the cells release determination revealed that D-limonene organogel-nanoemulsion with nisin demolished the integrity of the cells’ membrane, then causing the death of the microorganisms. This current study combined the advantages of organogel technology and the synergistic effects of multiple antimicrobial compounds in one system, not only widening the antimicrobial spectrum of nisin but also obviously reducing the amount of D-limonene and nisin. The combined use of D-limonene and nisin may provide a label friendly alternative to some of the currently used methods. The use of D-limonene organogel-nanoemulsion containing nisin as an antimicrobial treatment is relatively new and requires further studies. The interaction between D-limonene organogel-nanoemulsion with nisin and various food compounds and matrices has to be studied to better understand the physiochemical properties of this system. With greater understanding of the system as a whole, the use of antimicrobial organogel-nanoemulsion may find a broad range of applications within the production, processing, service and consumption of foods and beverages.

## Conflict of Interest Statement

The authors declare that the research was conducted in the absence of any commercial or financial relationships that could be construed as a potential conflict of interest.
